# Developmental Analysis of *Mimulus* Seed Transcriptomes Reveals Functional Gene Expression Clusters and Four Imprinted, Endosperm-Expressed Genes

**DOI:** 10.3389/fpls.2020.00132

**Published:** 2020-02-25

**Authors:** Miguel A. Flores-Vergara, Elen Oneal, Mario Costa, Gonzalo Villarino, Caitlyn Roberts, Maria Angels De Luis Balaguer, Sílvia Coimbra, John Willis, Robert G. Franks

**Affiliations:** ^1^ Department of Plant and Microbial Biology, North Carolina State University, Raleigh, NC, United States; ^2^ Department of Biology, Duke University, Durham, NC, United States; ^3^ GreenUPorto, Sustainable Agrifood Production Research Centre, Biology Department, Faculty of Sciences, University of Porto, Porto, Portugal; ^4^ Biology Department, San Diego State University, San Diego, CA, United States; ^5^ Department of Biology, Berea College, Berea, KY, United States

**Keywords:** seed development, ribonucleic acid sequencing, developmental time course analysis, endosperm, genomic imprinting, K-means clustering, MADS-box genes, *Mimulus guttatus*

## Abstract

The double fertilization of the female gametophyte initiates embryogenesis and endosperm development in seeds *via* the activation of genes involved in cell differentiation, organ patterning, and growth. A subset of genes expressed in endosperm exhibit imprinted expression, and the correct balance of gene expression between parental alleles is critical for proper endosperm and seed development. We use a transcriptional time series analysis to identify genes that are associated with key shifts in seed development, including genes associated with secondary cell wall synthesis, mitotic cell cycle, chromatin organization, auxin synthesis, fatty acid metabolism, and seed maturation. We relate these genes to morphological changes in *Mimulus* seeds. We also identify four endosperm-expressed transcripts that display imprinted (paternal) expression bias. The imprinted status of these four genes is conserved in other flowering plants, suggesting that they are functionally important in endosperm development. Our study explores gene regulatory dynamics in a species with *ab initio* cellular endosperm development, broadening the taxonomic focus of the literature on gene expression in seeds. Moreover, it is the first to validate genes with imprinted endosperm expression in *Mimulus guttatus*, and will inform future studies on the genetic causes of seed failure in this model system.

## Introduction

Upon their emergence in the Early Cretaceous, seed-bearing plants diversified rapidly, displacing older plant lineages and colonizing nearly every terrestrial habitat (Lidgard and Crane, 1988; Crane and Lidgard, 1989; Magallón and Castillo, 2009). Among the many factors enabling their swift rise to dominance was the emergence of seeds, a major evolutionary and reproductive innovation that frees vascular plants from a dependence on water for gametophytic dispersal and enables the next sporophytic generation to delay germination until conditions are favorable for growth and reproduction. Angiosperm seeds are formed by a unique process called double fertilization, wherein two haploid sperm nuclei contained within a pollen grain act separately to fuse with the haploid egg cell and the homo-diploid central cell of the female megagametophyte to form a diploid embryo and triploid endosperm.

Following double fertilization, angiosperm seeds undergo processes of cell differentiation, patterning, and growth. Early embryogenesis establishes the basic shoot-root body plan, after which the embryonic tissue and major organs of the embryo are formed by morphogenesis (West and Harada, 1993; Jürgens et al., 1994). Endosperm development in modern plants can be characterized by three major types: *ab initio* cellular, where each nuclear division is accompanied by cell division; nuclear, where a syncytial phase of free nuclear division is followed by cellular wall formation; and helobial, where an initial division of the primary endosperm cell results in two regions, at least one of which will exhibit free nuclear development (Friedman, 1994; Friedman, 2001). Cellular endosperm development is found in several basal angiosperm lineages (Friedman, 2001) and many diverse groups of asterids, and has likely evolved multiple times independently (Geeta, 2003). As the seed matures the endosperm will accumulate storage reserves for nutritional support of the mature embryo. Once fully formed, the mature embryo will enter a period of developmental arrest in preparation for dormancy.

Coordinated development between seed tissues is critical to ensuring normal development and plays a major role in determining the size of mature seeds (Garcia et al., 2003; Ingouff et al., 2006; Sechet et al., 2018), and recent improvements in transcriptomics have greatly improved our knowledge of the gene expression dynamics involved (Belmonte et al., 2013). In *Arabidopsis,* many genes are seed specific, including transcription factors (TFs) that regulate gene networks involved in cell differentiation and nutrient storage (Le et al., 2010; Chen et al., 2014; Yi et al., 2019). A subset of genes expressed in endosperm exhibit imprinted expression, an epigenetic phenomenon whereby alleles are differentially expressed depending upon their parent-of-origin (Grossniklaus et al., 1998; Hsieh et al., 2011; Luo et al., 2011; Waters et al., 2013; Florez-Rueda et al., 2016; Zhang et al., 2016; Lafon-Placette et al., 2018).

To date, most studies on gene expression in seeds have focused on a taxonomically narrow group of species with nuclear endosperm, such as *Arabidopsis* and crops from the family Poaceae. There exists a lack of studies illustrating the transcriptional dynamics of seeds with cellular endosperm development despite the prevalence of this developmental phenotype. We fill this gap by characterizing the gene expression dynamics associated with seed development in *Mimulus*, an asterid which exhibits *ab initio* cellular endosperm development. We perform a time series RNA sequencing experiment to illustrate the major transcriptional events associated with early seed development. Our work has two major goals: first, by focusing on a phylogenetically divergent species with cellular endosperm development it will serve as a data resource enabling comparative studies of gene expression in seed plants. Second, because hybrid seed inviability is a common outcome of hybridization and may be a major cause of speciation in plants, including *Mimulus*, characterizing the transcriptional dynamics of normally developing seeds will provide context for future studies on gene expression changes associated with hybrid seed lethality. Imprinted loci may play key roles in the proper development of endosperm (Grossniklaus et al., 1998) and divergence in the imprinting status of genes has been linked to the rapid emergence of reproductive barriers between even closely related species (Florez-Rueda et al., 2016; Lafon-Placette et al., 2018) including *Mimulus* (Kinser et al., 2018). Thus, although our study design prohibits a systematic screening for imprinted loci, we examine our data for genes exhibiting paternally imprinted expression in endosperm, as such genes have been implicated in the emergence of hybrid seed inviability. We identify genes whose expression exhibits significant temporal changes associated with key developmental shifts and characterize their patterns of co-expression and biological functions using K-means clustering and gene ontology enrichment analyses. We also identify and validate four genes (*ATRX5, MBD13, DnaJ, BGAL11*) that are paternally imprinted and whose homologues are imprinted in other plants, but which are not linked to hybrid endosperm failure in *Mimulus* (Kinser et al., 2018) or other taxa (Hatorangan et al., 2016; Florez-Rueda et al., 2016; Lafon-Placette et al., 2018). Our study represents an important first step in illustrating the gene regulatory dynamics of *Mimulus* seeds, is the first to identify and validate genes with imprinted endosperm expression in *Mimulus guttatus*, and will inform future studies on the genetic causes of seed failure in this model system.

## Materials and Methods

We examined hybrid seed from a compatible cross between two members of the *M. guttatus* complex: a serpentine-adapted annual *M. guttatus* and *Mimulus pardalis*, a facultatively selfing annual that is fully interfertile with the outcrossing *M. guttatus. M. pardalis* seed was collected in 2005 near the Star-Excelsior Mine in Copperopolis, CA (−120.856W, 38.153N). *M. guttatus* seed was collected in 2008 from the Donald and Sylvia McLaughlin Natural Reserve in Lake County, CA (−122.415W, 38.861N). Inbred lines from each population were formed by a minimum of five generations of selfing and are named SEC39 (*M. pardalis*) and CSS4 (*M. guttatus*), leading to an expectation of a maximum of 3% genomic-wide heterozygosity in each line. All individuals used here for morphological analyses as well as DNA and RNA sequencing are from these inbred lines. We chose these two populations because they are interfertile (based on our prior unpublished work and Macnair and Cumbes (1989)) but sufficiently genetically distinct to allow us to distinguish the allelic origin of genic SNPs, and thus search for genes that potentially exhibit *M. guttatus*-bias. All seeds were cold-stratified for 10 days at 4°C before being transferred to a growth chamber at 18-h days, 21°C, and 30% relative humidity. Crosses and self-pollinations were performed as described previously (Oneal et al., 2016).

### Seed Histology

We characterized the morphology and developmental ontogeny of selfed and hybrid seeds of *M. pardalis* and *M. guttatus* in order to provide a developmental framework for our time series analysis of gene expression. All fruits were collected from flowers that had been emasculated 1–3 days prior to fertilization. We used LR-white embedding to visualize and categorize the morphological progression of seed development. Ovaries and fruits were harvested at 0, 2, 4, 6, and 8 days after pollination (DAP) and vacuum-fixed in a solution of 2% paraformaldehyde, 2.5% glutaraldehyde, and 0.001% Tween 20 in 0.025 M PBS (pH 7), then incubated overnight at 4°C. Tissue was washed with 0.025 M PBS for 20 min, then dehydrated in an ethanol series (25%, 35%, 50%, 70%, 80%, 90%, and 3x 100%). LR-white (London Resin Company Ltd, London, UK) impregnation was performed by incubation for 12 h in increasing concentration of resin (10%, 25%, 50%, 75%, 90%, and 2x 100%). Samples were transferred to gelatin capsules and allowed to polymerize for 48 h in a 56°C oven. The 2-micron sections were obtained with a Leica EM UC7 Ultramicrotome, placed on glass slides, and stained with 1% toluidine blue followed by 1% safranin.

### Preparation of Libraries and Messenger RNA Sequencing

For the purpose of transcriptomic analysis, total RNA was extracted from ovules and developing hybrid seeds from the SEC39 x CSS4 cross (throughout this paper, the maternal parent is listed first in cross notations). We only prepared a single direction of this cross for transcriptomics analysis due to a significant level of pre-zygotic infertility in the CSS4 x SEC39 crossing direction, which limited the RNA available for sequencing. Ovules/seeds were released from the ovary/fruit by gently shaking into microcentrifuge tubes containing 300 μl of ice-cold isolation buffer [1X First-Strand buffer Invitrogen, 1 mM dithiothreitol (DTT), and 4% RNaseOUT]. TRIzol was then added (3:1 ratio of TRIzol:isolation buffer). We used the Pure Link RNA Mini Kit (Invitrogen), modifying it as follows: collected ovules and seeds in solution were ground gently with pellet pestles in Eppendorf tubes for 2–4 min then mixed with 240 μl of chloroform. The solution was shaken vigorously by hand for 15 s, then incubated for 3 min at room temperature followed by centrifugation at 12,000*g* for 15 min at 4°C to separate phases. Total RNA was isolated according to the manufacturer’s instructions, then treated with TURBO DNA-free Dnase I Kit (Ambion) for removal of contaminated DNA. The mixed solution was then spun at 10,000*g* for 1.5 min to collect the top layer of RNA. We note that this method of hand separation of ovules/seeds from the longitudinal-sectioned fruits may have inadvertently resulted in a small amount of placental-contributed tissue into our samples.

We generated and sequenced a total of 20 RNA sequencing libraries (see **Table 1** for details). We used four maternal plants (SEC39) and five pollen donors (CSS4) to generate hybrid seeds aged 2, 4, 6, and 8 days after pollination (DAP). Because of the difficulty of isolating sufficient RNA for sequencing, ovules were derived from seven maternal plants. Hybrid seed pools generated by one maternal plant constituted one biological replicate. Because our parental plants were highly inbred, allelic differences between replicates should be minimal. This expectation is confirmed by the genomic sequence data (see below), which indicates that in CSS4, 0.115% of covered exonic sites are heterozygous, while in SEC39, 0.04% covered exonic sites are heterozygous. The RNA integrity number (RIN) for all samples > 7.8, as determined by an Agilent 2100 Bioanalyzer. Twenty strand-specific complementary DNA (cDNA) libraries were prepared from approximately 100 ng of total RNA by the North Carolina State University Genomic Science Laboratory using a NEB Ultra Directional Library Prep Kit for Illumina then sequenced on an Illumina NextSeq 500. Average size of the cDNA fragments was approximately 380 bp.

**Table 1:** Biological replicates of SEC39 ovules and SEC39 x CSS4 hybrid seeds.

Time point (DAP)	Biological replicates	No. maternal plants	No. pollen donors	Sample names
0	5	8	N.A.	0d_BR1_S1, 0d_BR4_S10, 0d_BR5_S15 0d_BR6_S17, 0d_BR7_S19
2	4	4	5	2d_BR1_S11, 2d_BR2_S16, 2d_BR3_S18 2d_BR4_S20
4	3	3	5	4d_BR2_S4, 4d_BR3_S7, 4d_BR4_S12
6	4	4	5	6d_BR1_S2, 6d_BR2_S5, 6d_BR3_S8, 6d_BR4_S13
8	4	4	5	8d_BR1_S3, 8d_BR2_S6, 8d_BR3_S9, 8d_BR4_S14

Altogether, we used eight maternal plants and five pollen donors. Maternal plants #1 and 4 were used to generate two ovule samples (0d_BR1_S1 and 0d_BR4_S10), and maternal plants 1–4 were used to generate all hybrid seeds collected from 2 to 8 days after pollination (DAP. Because of the difficulty of isolating sufficient RNA for sequencing of unfertilized ovules, we pooled samples derived from maternal plants 2 and 3 with samples from three additional maternal plants not used elsewhere in this study to generate samples 0d_BR5_S15, 0d_BR6_S17, and 0d_BR7_S19. The same five pollen donors were used to generate all hybrid seeds.

### RNA Sequence Alignment Strategy

Known pairwise polymorphism in the *M. guttatus* complex ranges from 0.033 to 0.065 (Brandvain et al., 2014; Gould et al., 2017), raising the prospect of bias from mapping RNA reads to the *M. guttatus* reference genome, which is likely to be different from the lines utilized here. To reduce the number of reads discarded during mapping, we used a pseudo-reference genome mapping approach, mapping our RNA reads to parental pseudo-reference genomes that we generated from whole-genome DNA sequence data from each parental inbred line. Sequence data was obtained from DNA extracted from bud tissue from one maternal parent and one pollen donor using the GeneJET Plant Genomic DNA Purification Kit (Thermo Fisher Scientific). Illumina libraries were prepared by the Duke University Genome Sequencing Facility using the Illumina TruSeq DNA Nano library prep kit, then sequenced on an Illumina HiSeq 4000 machine, generating 150 bp paired-end reads. We removed adaptors and low quality reads with Trimmomatic (Bolger et al., 2014) and mapped the remaining reads using bwa mem (http://bio-bwa.sourceforge.net), retaining only properly paired reads. Mean coverage was 38x and 32x for CSS4 and SEC39, respectively. Variants were called using GATK3.7 and hard-filtered by quality-by-depth (< 2.0), strand bias (> 60.0), mapping quality (< 20.0), mapping quality rank sum (< 12.5), and the read position rank sum test (< −8.0). We also removed variants with low coverage (< 4) and high coverage (> 2 SD from the mean). We generated parental pseudo-reference genomes that incorporated our filtered variants using the package ModTools (Huang et al., 2014). We used bedops (Neph et al., 2012) to calculate pairwise coding sequence divergence between SEC39 and CSS4.

We mapped RNA reads to each parental pseudo-reference using the default settings of the splice-aware aligner STAR, allowing a maximum number of mismatches relative to read length of 0.04 (Dobin et al., 2013). We then used the lapels/suspenders pipeline (Huang et al., 2014; Crowley et al., 2015) to filter reads by mapping position and quality. For each read, lapels determines its mapping location in the maternal and paternal pseudo-reference, compares their mapping qualities in each (as determined by the number of mismatches), and then selects the mapping position with the highest quality. For our study, reads that mapped uniquely to only one parental genome were retained, while reads that mapped to both parental genomes were assigned the coordinates with the highest mapping quality. Only properly paired, uniquely mapped reads were retained for downstream analyses.

### Time Series Analysis of Gene Expression Changes With Development

We generated gene counts on our lapels/suspenders filtered alignments using featureCounts (Liao et al., 2014). We performed a principle component analysis (PCA) of normalized gene expression estimates from the 20 RNA libraries using the PlotPCA function of DESeq2, using the 500 most highly variable genes (Love et al., 2014). We took two approaches to discovering genes that were differentially expressed over the course of seed development. First, we combined multiple pairwise comparisons (0 *vs*. 2 DAP, 2 *vs*. 4 DAP, 4 *vs*. 6 DAP, and 6 *vs*. 8 DAP) into a generalized linear model in edgeR, performing a likelihood ratio test to determine significance with a false discovery rate (FDR) of 0.01. Count data were trimmed mean of M values (TMM)-normalized and only genes with greater than 1 count-per-million in at least three samples were retained (Chen et al., 2016). Second, we used Next maSigPro, a program which employs a least-squared polynomial regression and log-likelihood ratio test to detect genes exhibiting significant changes in expression over time (Conesa et al., 2006; Nueda et al., 2014). Next maSigPro selects the best model by goodness-of-fit using a correlational cutoff (i.e., R^2^ value) supplied by the user. Such time series models are an improvement over performing multiple pairwise comparisons (Spies et al., 2019), especially when several time points are sampled. Next maSigPro includes batch effects in its regression model; we considered each maternal parent to be a batch, giving us seven total batches: four maternal parents distributed across 0–8 DAP and three additional batches corresponding to the three pooled samples from 0 DAP. We performed a polynomial regression with an R^2^ of 0.6 and Benjamini-Hochberg corrected FDR of 0.01. We took the overlap between edgeR and the Next maSigPro time series analysis to be the set of genes that were *differentially expressed* over the course of seed development. All further downstream analyses were performed on these differentially expressed genes (DEGs). We validated our RNA isolation, mapping, and normalization of gene expression by performing quantitative real-time PCR (qRT-PCR) with independently-collected, triplicate RNA samples from unfertilized ovules and whole hybrid seeds from a new set of three maternal plants and three pollen donors on a subset of six genes (see **Figure S1** for details) and comparing their trends to the transcriptomics analysis. The qRT-PCR temporal expression patterns of all six genes closely matched the RNA-seq based expression patterns.

To uncover trends in the gene ontology (GO) terms of DEGs, we performed the Hartigan and Wong K-means clustering algorithm (Hartigan and Wong, 1979) on the fragments per kilobase of transcript per million (FPKM) values of all DEGs. We used 20 clusters (as determined by the gap statistic), with 100 random starts and 25 iterations. We performed a GO-term enrichment analysis of resulting clusters in ThaleMine (https://apps.araport.org/thalemine/begin.do) using the most similar *Arabidopsis thaliana* homologues, then used REVIGO (http://revigo.irb.hr/) to filter out redundant GO-terms, using a similarity cutoff index of 0.7. While we used the reference annotation (Phytozome v2.0) to calculate *Mimulus* gene abundances, we established homology of genes relative to *Arabidopsis* using BLASTx (Camacho et al., 2009) with a cutoff of E ≤ 0.001, designating the hit with the highest bitscore as the most similar *A. thaliana* homolog. There were few differences between the BLASTx results and the phytozome annotation (Goodstein et al., 2012). Here and elsewhere in this paper, we present the gene name and gene designation for the most similar homologue from *Arabidopsis thaliana* for the *M. guttatus* gene number. We used plantTFDB 4.0 (http://planttfdb.cbi.pku.edu.cn/) to annotate transcription factors in *M. guttatus*, tomato (*Solanum lycopersicum*), *A. thaliana*, and rice (*Oryza sativa*) (Jin et al., 2015; Jin et al., 2017). We aligned protein sequences of type I MADS-box genes from these species, and estimated and bootstrapped a neighbor joining (NJ) tree (1000 reps) using clustalW2.1 (Larkin et al., 2007).

We compared our expression data with previously published *M. guttatus* transcription data collected from calyx, leaves, petals, and stem tissue (Edger et al., 2017) to determine which genes and transcription factors are expressed exclusively in ovules or seeds. We blasted the transcripts in Edger et al. (2017) to the *M. guttatus* reference genome to obtain their gene identity, selecting the hit with the highest bitscore and using a cutoff of E ≤ 0.001. We note that both our data and that of Edger et al. (2017) include transcripts that do not correspond to the current *M. guttatus* genome annotation; we chose not to pursue these unannotated transcripts further. We considered a gene to be *expressed* in our data if it had a mean FPKM ≥ 1.0 in at least one time point. We categorized as *stage-specific* those genes whose expression in our samples had a mean FPKM ≥ 5.0 at that stage but ≤ 1.0 at all other stages. Finally, we categorize genes as *exclusive* to ovules or seeds if their expression levels as reported in Edger et al. (2017) was an FPKM < 1.0 and they had a minimum mean expression of FPKM ≥ 5 in ovules or 2–8 DAP seeds. We compare our data to seed transcriptomes of *A. thaliana* (Belmonte et al., 2013), maize (Chen et al., 2014; Yi et al., 2019), and domesticated tomato and a near wild relative (*Solanum pimpinellifolium*) (Pattison et al., 2015; Shinozaki et al., 2018) to look for overlapping sets of *seed-exclusive* genes in these taxa.

### Detecting and Validating Genes With *Mimulus guttatus*-Biased Expression

We used GATK’s ASEReadCounter to assemble *M. guttatus* and *M. pardalis* allele counts at SNP positions distinguishing our inbred lines, CSS4 and SEC39, for each library of 2–8 DAP seeds. We confined our allele counts to sites that were homozygous in the 5G inbred lines used to generate the parental pseudo-references. At each time point, we assembled lists of genes that either 1) exhibited no maternal expression at any SNP in any biological replicate, or 2) exhibited significant paternal bias for one or more SNPs within a gene for two or more replicates. For the former we eliminated genes exhibiting either little overall expression (< 2 counts averaged across replicates) or whose expression across replicates within a time point was highly variable (i.e., standard deviation in expression > mean expression). For the second group, at each SNP we calculated the ratio of *M. guttatus* allelic counts to *M. pardalis* allelic counts (Mg/Mp) for each replicate, then calculated the mean Mg/Mp ratio across replicates. We retained only those genes where a majority of SNPs within the gene exhibited Mg/Mp > 2 and where variance in Mg/Mp was low (i.e., where the standard deviation of Mg/Mp among SNPs was less than the mean across all SNPs within a gene). Only genes expressed in two or more replicates were considered for future validation. Because our search for *M. guttatus*-biased expression was performed on whole-seed transcriptomes, we cannot apply any *a priori* hypothesis for the ratio of Mg/Mp expression at any given gene, since that gene may be expressed in one or more tissue types (i.e., seed coat, embryo, and/or endosperm). Thus, our findings do not constitute a screening for imprinted genes in *Mimulus*.

Transcripts that display an expression bias toward the *M. guttatus* allele could originate from a parent-of-origin bias, an allele-of-origin bias or be false positives. To both validate the expression bias and distinguish between these possibilities we hand-isolated endosperm from both SEC39 x CSS4 and CSS4 x SEC39 F1 seed at 8 DAP. RNA was converted to cDNA and used in semi-quantitative PCR assays. We identified seven candidate genes containing known allele-distinguishing SNPs due to the creation or elimination of a restriction enzyme cutting site. We created pairs of oligos (**Table S1**) to amplify the SNP-containing fragments. Relative expression of maternal and paternal alleles was assayed by agarose-gel electrophoresis and by Sanger sequencing.

## Results

We generated 20 RNAseq libraries with a total of 3.85 x 10^8^ raw reads, averaging 1.93 x 10^7^ reads per library (see **Table S2** for alignment statistics). Pairwise sequence divergence (π) between SEC39 and CSS4 in coding regions is 0.028 ± 0.022 S.D. Mapping to the SEC39 pseudoreference produced a mean of 75.3% ± 4.25 S.D. properly paired, uniquely mapped reads, while mapping to the CSS4 pseudoreference produced a mean of 70.0% ± 2.34 S.D. properly paired, uniquely mapped reads. Mapping to the SEC39 (i.e., maternal) pseudoreference genome was more efficient since our samples contain reads from seed coat (all maternal), embryo (1:1 maternal:paternal), and endosperm (2:1 maternal:paternal). Altogether, our merged alignments have an average of 75.5% ± 3.98 S.D. paired, uniquely mapped reads. This represents a significant improvement over mapping to the IM62 reference genome, which produced 67.7% ± 4.0 S.D. uniquely mapped reads (t_19__=_ 68.97, p < 0.0001) for an increased mapping rate of ~8%, similar to that found by others (Huang et al., 2014; Crowley et al., 2015).

### Developmental Progression of *Mimulus pardalis* x *Mimulus guttatus* Seeds

**Figure 1:**
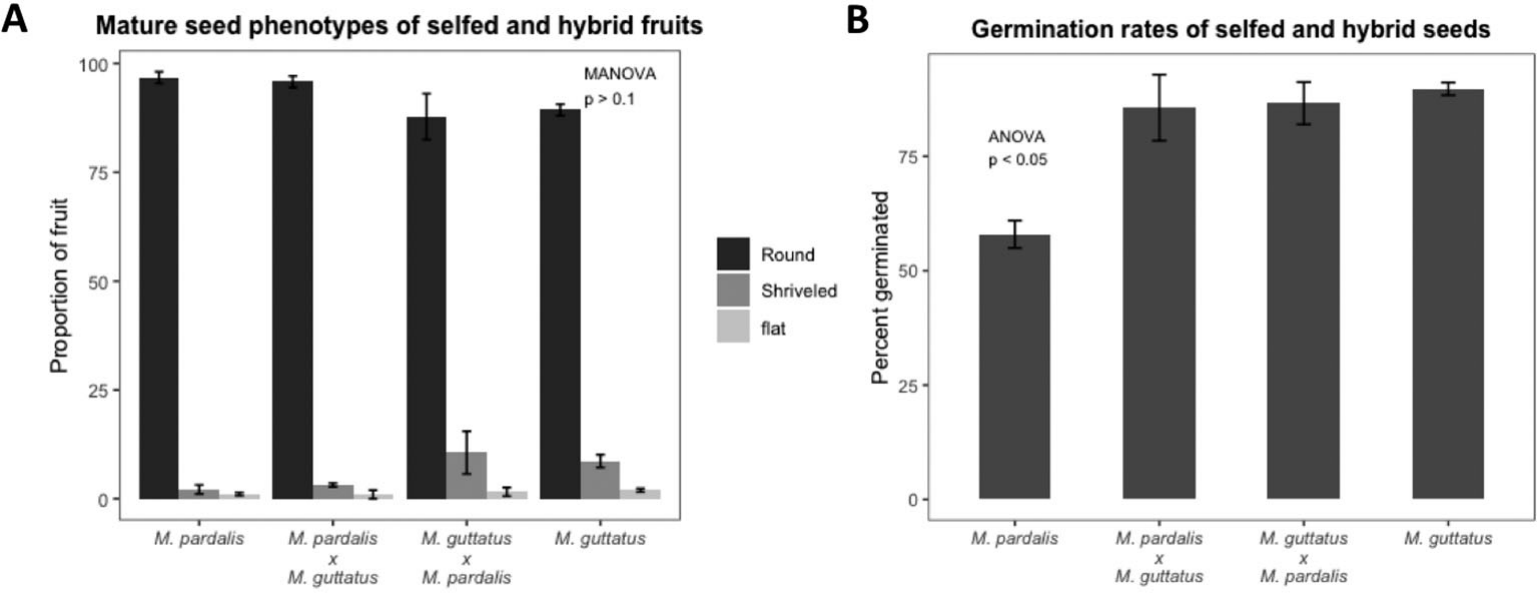
**(A)** Percentage of mature seed phenotypes (round, shriveled, and flat) recovered from fully developed fruits from selfed *Mimulus pardalis* and *Mimulus guttatus* and reciprocal crosses of *M. pardalis* x *M. guttatus.* There was no significant difference in the distribution of seed types between the selfed fruits and hybrid fruits (MANOVA F_9,45_ = 1.09, p > 0.1). **(B)** Germination rates of selfed *M. pardalis* and *M. guttatus* seed, as well as *M. pardalis* x *M. guttatus*, and *M. guttatus* x *M. pardalis* hybrid seeds. Mp, *M. pardalis*; Mg, *M. guttatus*. Bars indicate standard error. Germination rates were generally high (57.9–89.74%) (N=4 for each selfing and cross).

As expected, seed set, outer morphology, and germination rates among mature seeds from *M. pardalis* x *M. guttatus* (SEC39 x CSS4) confirms a lack of postzygotic isolation between these inbred lines (Oneal, Munger and Willis, unpublished; Macnair and Cumbes (1989)) (see **Figure 1**, **Figure S2**). Selfed *M. pardalis,* selfed *M. guttatus,* and reciprocal *M. pardalis* x *M. guttatus* fruits contained mostly round, viable seeds with a small minority of shriveled or flat seeds (**Figure 1A**, **Figure S2**), with no significant difference in the distribution of seed types between the selfed fruits and hybrid fruits (MANOVA F_9,45_ = 1.09, p > 0.1). Germination rates were generally high (57.9–89.74%) (**Figure 1B**).

**Figure 2:**
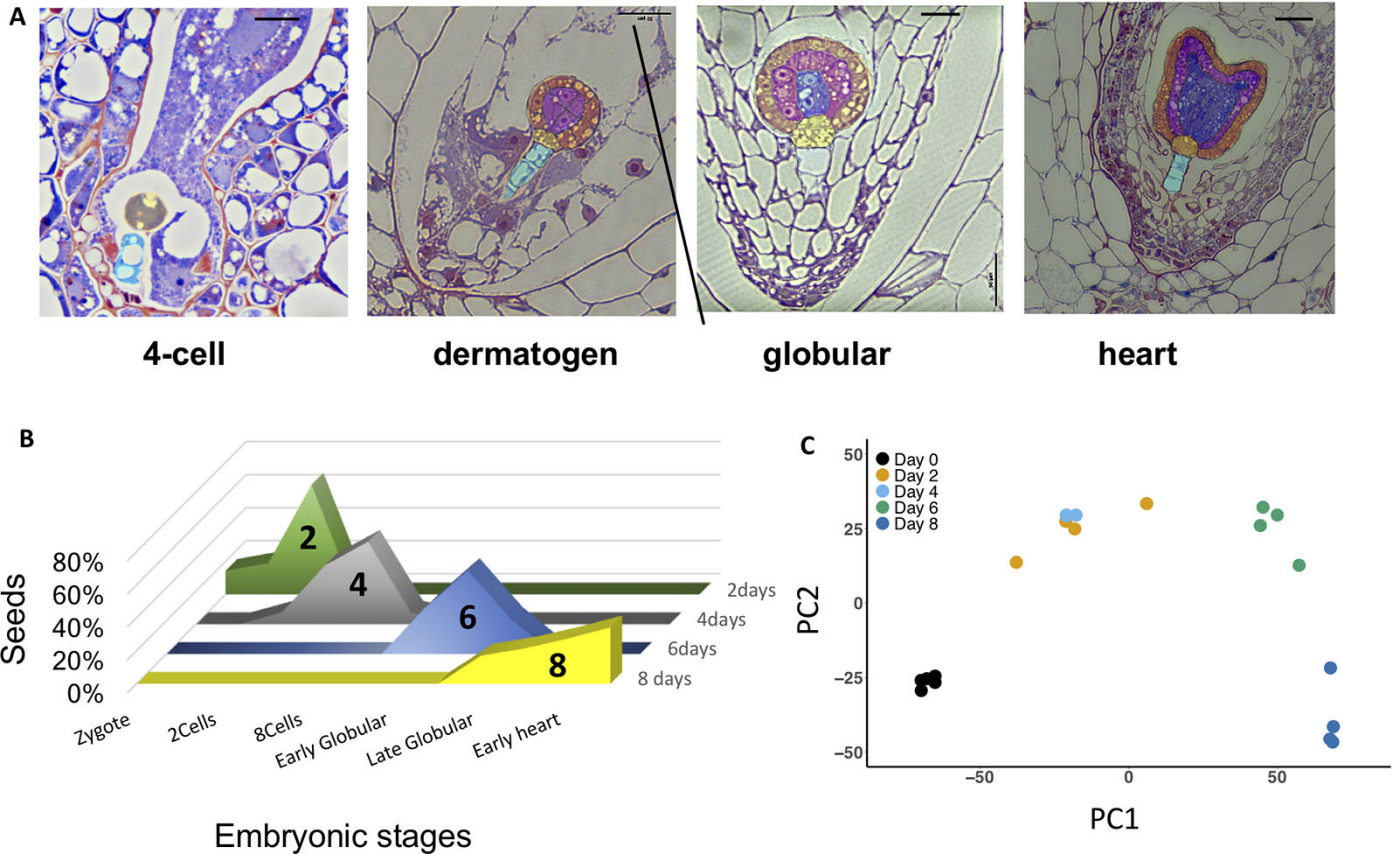
**(A)** Morphology of *Mimulus pardalis* x *Mimulus guttatus* developing seeds collected 2, 4, 6, and 8 days after pollination (DAP). Sections were obtained from LR-white embedded seeds (see *Methods*). **(B)** Frequency distribution of embryonic stages of seeds collected at 2, 4, 6, and 8 DAP. **(C)** Principle component analysis (PCA) plot of RNA libraries.

At 2 DAP, most seeds contain four-cell embryos, progressing to eight-cell and dermatogen embryos by 4 DAP (**Figure 2A**). By 6 and 8 DAP, seeds contain late globular- and heart-stage embryos, respectively. Endosperm development is *ab initio* cellular (Arekal, 1965; Oneal et al., 2016). There is regular proliferation of endosperm between 4 and 8 DAP, and limited variation in the distribution of embryonic stages at each time point (**Figure 2B**), which may result from differences in the rate of pollen tube growth and timing of fertilization. A PCA of normalized gene expression values from the 20 libraries produced well-defined clusters corresponding to the five different developmental time points assayed (**Figure 2C**), with the exception of some overlap between 2 and 4 DAP, indicating that these stages exhibit similar transcriptional states. The first axis explains 66% of the variation, and reflects the effect of time, while the second axis explains 22% of the variation.

### Correlations Between Gene Expression and the Progression of *Mimulus guttatus* Seed Development

We detected the expression of 18,836 annotated genes in ovules and seeds, representing 67% of all currently annotated *M. guttatus* genes, including 1,011 transcription factors (TFs), of which 40 are MADS-box genes. The number of genes expressed in any stage was highest in ovules (16,681) and lowest in heart-stage seeds (8 DAP: 14,784), but we found no relationship between gene expression diversity and collection time point (ANOVA F_4,15_ = 2.522, p = 0.085) (**Figure S3**). A generalized linear model incorporating multiple, progressive pairwise comparisons in edgeR identified 12,384 DEGs (FDR ≤ 0.01), while a time series analysis in Next maSigPro identified 6,613 DEGs (R^2^ = 0.6, FDR ≤ 0.01) (**Tables S3** and **S4**). We focus on the overlapping sets of DEGs between edgeR and Next maSigPro. These consisted of 6,233 DEGs (**Figure S4**), including 388 transcription factors (TFs) from 49 families, of which 16 are type I MADS and 4 are MIKC-type MADS-box genes.

### Clustering Reveals Co-Expression Patterns Enriched for Functional Pathways

**Figure 3:**
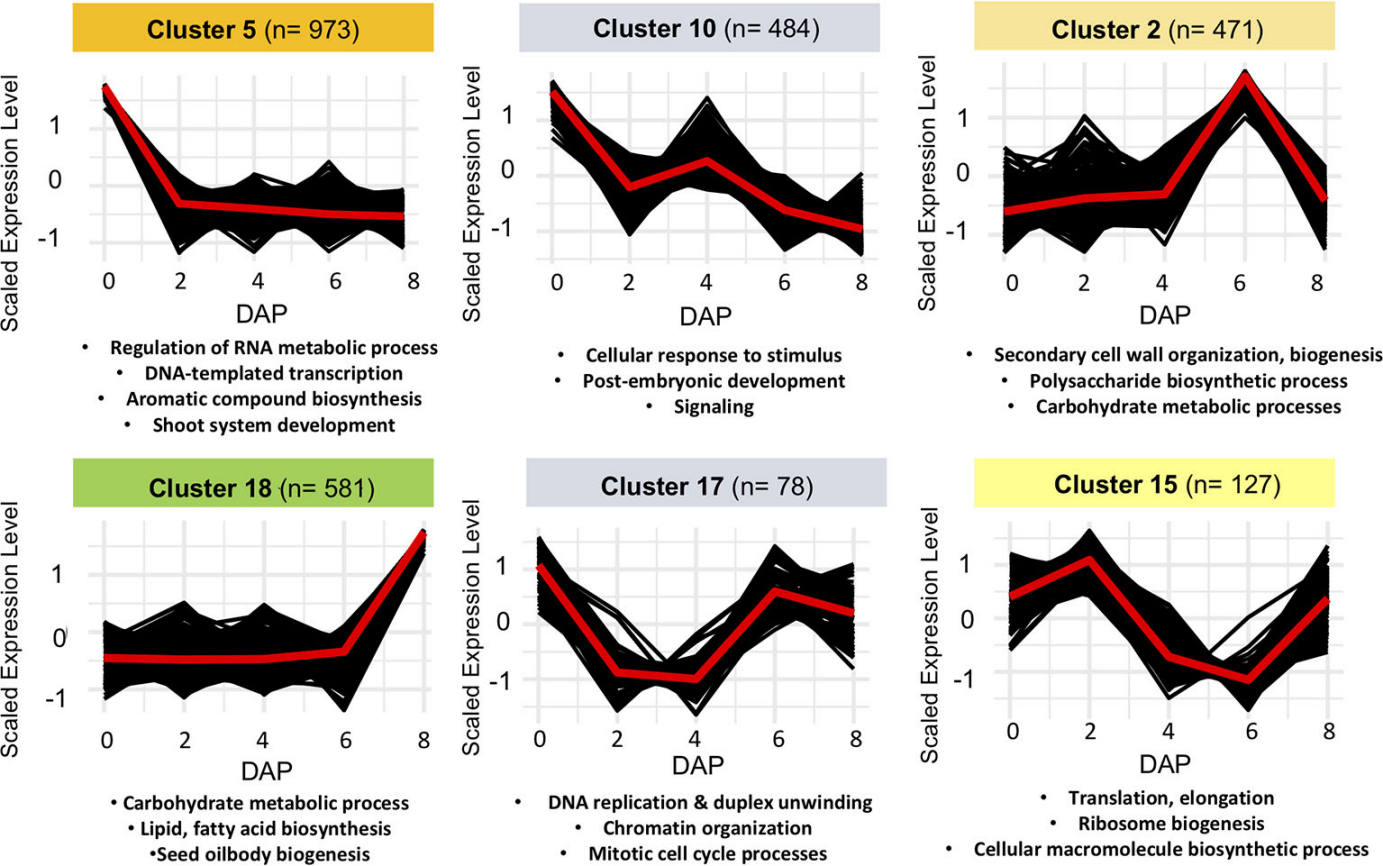
Examples of non-overlapping k-means clusters of co-expressed genes and their significant gene ontology (GO) enrichment terms.

To further correlate patterns of gene expression with developmental changes in seeds, we used a Hartigan and Wong K-means clustering algorithm (Hartigan and Wong, 1979) on the 6,233 DEGs identified by edgeR and Next maSigPro. Of 20 K-clusters, 13 were significantly enriched for GO-terms, including several enriched for GO-terms known to play a role in seed development in *Arabidopsis* (see **Figure 3**, **Figure S5**, **Tables S5** and **S6**). Several of these clusters are of note and mirror what is known about gene expression patterns in developing seeds in other taxa. For example, genes that exhibit peak expression in ovules are enriched for regulation of RNA metabolic process, regulation of gene expression and transcription, and regulation of multicellular organismal development (cluster 5). In maize, genes that are highly expressed in the female gametophyte include transcription factors involved in RNA regulation (Chen et al., 2014). In *Arabidopsis*, epigenetic regulatory pathways established in the egg and central cell of the female gametophyte set the stage for the production of embryo-targeting small interfering RNAs (siRNAs) in the endosperm (Wuest et al., 2010). Development at 6 DAP is characterized by the emergence of the late globular-stage embryo (**Figure 2A**) and the acceleration of endosperm proliferation (Oneal et al., 2016). A group of co-expressed genes which peaks in both ovules and in globular (6 DAP) seeds is enriched for GO-terms related to cell cycle processes, DNA replication, and chromatin organization (cluster 17) as it is in *Arabidopsi*s (Le et al., 2010; Belmonte et al., 2013). Another cluster that peaks at 6 DAP (cluster 2) contains genes involved in cell wall biogenesis and organization. Finally, genes that remain unexpressed until 8 DAP are enriched for carbohydrate and lipid metabolic processes, seed oil body biogenesis, and lipid localization (cluster 18). In *Arabidopsis* and maize these biological processes are enriched in endosperm (Belmonte et al., 2013; Li et al., 2014), suggesting they may perform similar roles in species with *ab initio* cellular endosperm development.

We identified a subset of genes whose expression was *stage-specific* in our data (see *Methods*), with 72 genes specific to ovules, and 3, 29, and 77 genes specific to 2, 6, and 8 DAP seeds, respectively (**Table S7**). There were no genes *specific* to 4 DAP seeds from. A comparison of our data with expression data from *M. guttatus* calyx, petal, leaf, and stem tissue (Edger et al., 2017) revealed 30 genes whose expression was exclusive to ovules (i.e., FPKM ≥ 5 in ovules and ≤ 1 in any other tissue), including 4 transcription factors (TFs) (**Table S7**). There were 478 genes *exclusively* expressed in seeds, including 35 TFs from multiple families and 6 type I MADS-box genes encoding transcription factors, with 2, 20, and 48 genes *exclusive* to the 2, 6, and 8 DAP stages of seed development, respectively (**Tables S8** and **S9**). A heatmap of expression of *seed-exclusive* transcription factors in *M. guttatus* shows the majority are not constitutively expressed, but rather, are chiefly expressed in 6 or 8 DAP seeds (**Figure S6**).

### Overlap Between *Seed-Exclusive Mimulus guttatus* Genes and *Seed-Exclusive* Genes in Other Taxa

K-means clustering of genes expressed in seeds reveals that a large fraction (N=120, or 25%) of *seed-exclusive* genes segregate into cluster 18. Another 101 (21.1%) of *seed-exclusive* genes co-segregate into cluster 2. Comparing *seed-exclusive* genes in *M. guttatus* to other taxa, including *A. thaliana,* maize, and tomato reveals some overlap. In *A. thaliana*, there are 43 seed-exclusive genes whose nearest homologs are also seed exclusive in *M. guttatus* (**Table S7**); one of these genes is enriched in the *A. thaliana* seed coat (At5g39130) (Belmonte et al., 2013). Also among the *M. guttatus seed-exclusive* genes are two that are not exclusive to *A. thaliana* seeds but do exhibit enriched expression in *A. thaliana* endosperm: At*AGL62* (At5G60440) and At*CYS5* (At5g47550), a putative phytocystatin expressed in seedlings (Song et al., 2017). At*AGL62* suppresses endosperm cellularization in *A. thaliana* and is a key regulator of endosperm development. The *seed-exclusive* expression pattern of 2 *M. guttatus* homologues of *AGL62* (Migut.B00708 and Migut.D01476) suggests that they play an important role during *Mimulus* seed development; however, without RNA sequencing of isolated seed tissues and additional functional analyses, we cannot yet determine the role of *M. guttatus AGL62* homologues. Finally, there are 25 genes enriched in *A. thaliana* endosperm that are not expressed in *M. guttatus* seeds, including three transcription factors (At*AGL87*, At*FIS2*, and At*FWA*).

In maize, there are 23 seed exclusive genes whose nearest homologs are also *seed exclusive* in *M. guttatus* (**Table S7**), 11 enriched in endosperm, and 2 enriched in the embryo (Chen et al., 2014; Yi et al., 2019). Of the seed-exclusive genes in *M. guttatus*, 54 genes have homologs that are not seed-exclusive but were found to exhibit enriched expression in the maize embryo, endosperm, or both, relative to other seed tissues (Chen et al., 2014). There are 250 genes enriched in maize endosperm whose homologs are not expressed at all in *M. guttatus* seeds, including 39 transcription factors such as transcription factors activating maize *ABI3* (*VP1)*, *AUX/IAA*, and two maize type I MADS genes (*MADS25* and *MADS27*). Another gene enriched in maize endosperm but which is not expressed in *M. guttatus* seeds is the maize *FIE1*, which is maternally imprinted and peaks in expression during the transition from mitotic cell division to endoreduplication in the endosperm (Hermon et al., 2007). Twenty *M. guttatus* genes have nearest homologs that are exclusively expressed in both *A. thaliana* and maize seeds (**Table S7**). There is, however, no overlap between genes unexpressed in *M. guttatus* seeds but enriched in *A. thaliana* or maize endosperm.

Finally, in domesticated tomato there are 8 seed-exclusive genes whose nearest homologs (25 genes) are seed exclusive in *M. guttatus* (Zuluaga et al., 2016; Shinozaki et al., 2018), one of which (*CYS5*) is enriched in the embryo of a wild relative of domesticated tomato (Pattison et al., 2015) (Solyc04g014780). Of the 25 seed-exclusive genes *M. guttatus* with homologs in tomato, 22 do not have seed-exclusive homologs in *A. thaliana* or maize. These include *M. guttatus* and tomato homologs of *LIPID TRANSFER PROTEINS 4* and *12* (*LPT4* and *LPT12*: Migut.M01356/Migut.M01355/Migut.M01350/Solyc05g053530), *PECTIN METHYLESTERASE INHIBITOR 2* (*PMEI2*: Migut.A00119/Migut.I00331/Solyc09g072950), and *VACUOLAR INHIBITOR OF FRUCTOSIDASE 1* (*VIF1*: Migut.M00087/Solyc09g072950). A GO-term enrichment analysis of these genes finds two overrepresented biological processes: negative regulation of catalytic activity, and negative regulation of molecular function (adjusted p-values 0.003 and 0.004, respectively).

### Type I MADS-Box Gene Expression

**Figure 4:**
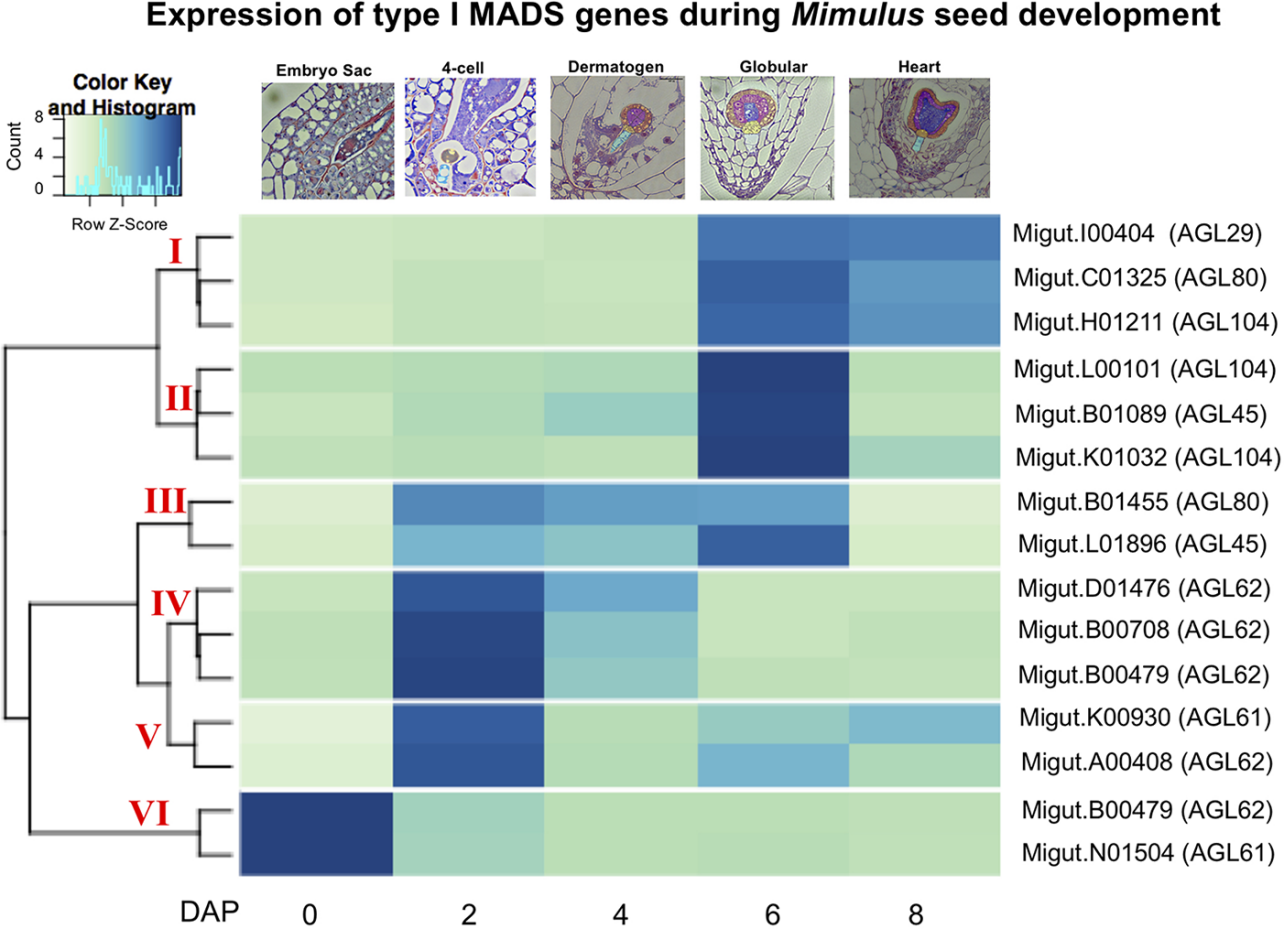
A heatmap of MADS-box type I gene expression in ovules and seeds. The nearest *Arabidopsis thaliana* homologue for each *Mimulus guttatus* MADS-box gene is indicated in parentheses (as determined by BLASTx).

**Figure 5:**
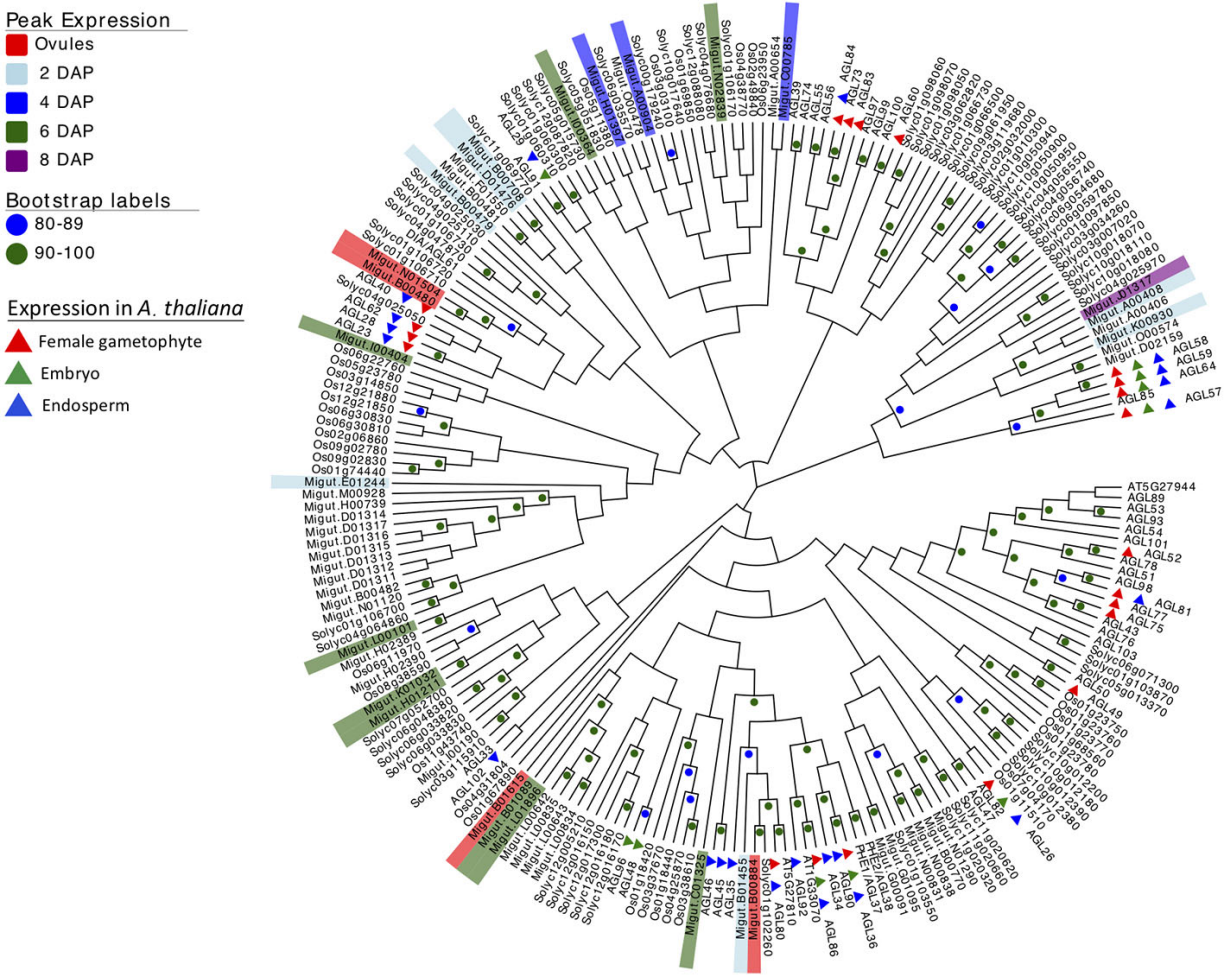
Neighbor joining tree of type I MADS-box genes from rice (*Oryza sativa*), *Arabidopsis thaliana*, tomato (*Solanum lycopersicum*), and *Mimulus guttatus* (reference line IM62). Bootstrap support is represented by color (green ≥ 90, blue 80–89). Nodes with less than 80% support are not marked. *M. guttatus* genes expressed in our data set are color-coded by time of peak expression. *A. thaliana* expression profiles are indicated by color-coded triangles.

Because of their known role in female gametophyte and seed development across a range of plant taxa (Colombo et al., 1997; Busi et al., 2003; Köhler et al., 2003; Arora et al., 2007; Colombo et al., 2008; Kang et al., 2008; Steffen et al., 2008; Zhang et al., 2018), we further examined the temporal expression patterns of differentially expressed type I MADS-box genes using a hierarchical clustering analysis. This analysis identified co-expressed sets, or hierarchical expression clusters of type I MADS box genes, a family that includes the *Arabidopsis AGAMOUS-LIKE* genes *AGL26*, *AGL62*, and *AGL104*. Two *Mimulus* type I MADS-box DEGs were expressed in ovules but unexpressed after fertilization (**Figure 4**, cluster VI); other MADS-box DEGs were either expressed primarily in four-cell to dermatogen-stage seeds (2–4 DAP, clusters IV and V), or in globular- and heart-stage seeds (6–8 DAP, clusters I and II) (**Figure 4**). A neighbor joining (NJ) tree of type I MADS genes from *M. guttatus*, *S. lycopersicum*, *A. thaliana*, and *O. sativa* suggests that in some cases, genes with similar temporal expression patterns are each other’s nearest relatives (**Figure 5**).

### Four Imprinted Endosperm-Expressed Genes Display Strong Paternal Expression Bias

**Figure 6:**
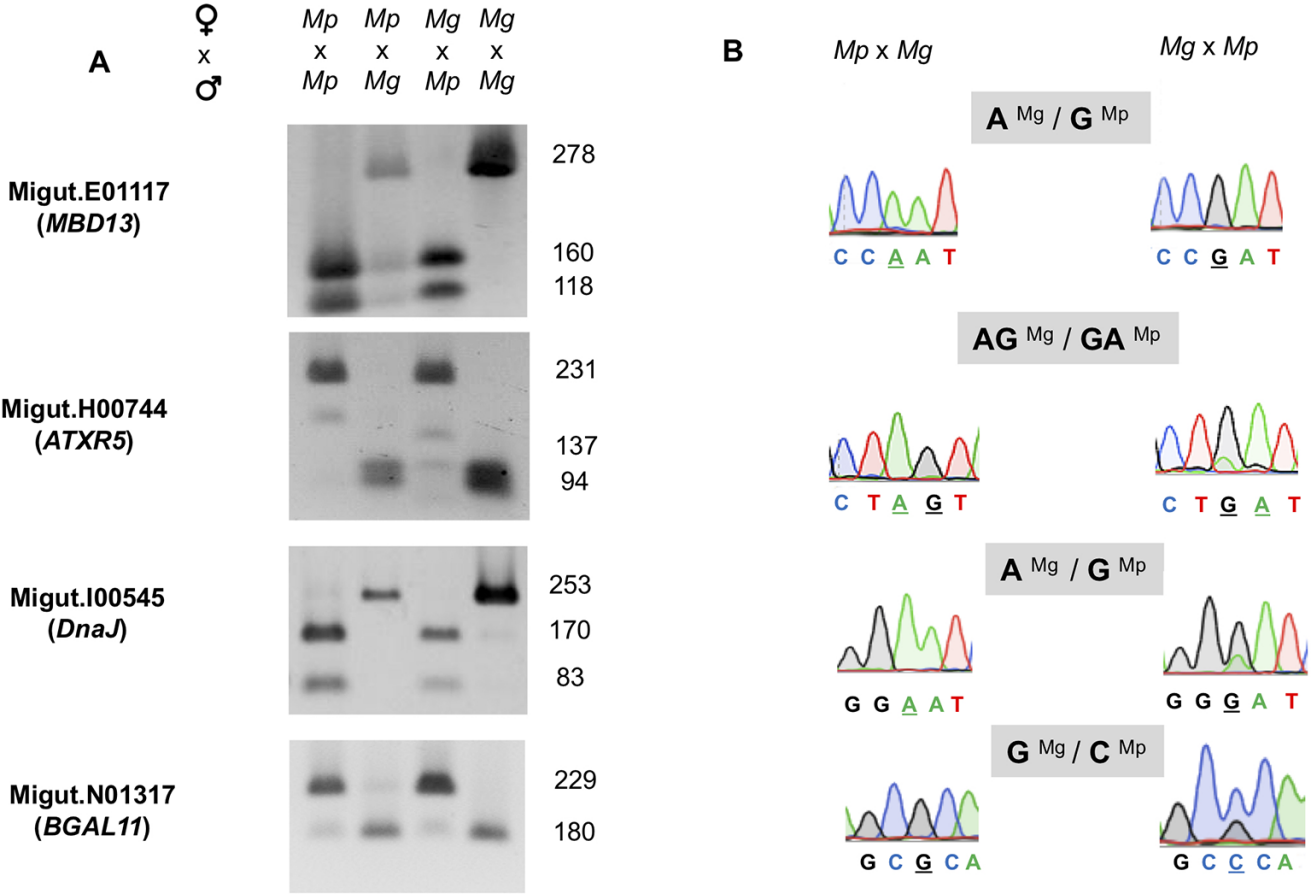
Validation of four paternally expressed imprinted genes: Migut.E01117 (*MBD13*), Migut.H00744 (*ATRX5*), Migut.I00545 (*DnaJ*), and Migut.N01317 (*BGAL11*) *via* semi-quantitative PCR and Sanger sequencing. **(A)** Allele-specific real-time (RT)-PCR analyses of each gene in the endosperm of selfed and reciprocal crosses of *Mimulus pardalis* (Mp) and *Mimulus guttatus* (Mg) is given. For each gene, gel shows the sizes of restriction fragments after RT-PCR amplification and digestion with restriction endonucleases. **(B)** Paternal expression was confirmed by real-time (RT)-PCR sequencing chromatographs at selected SNP regions measuring allele-specific expression in reciprocal crosses.

We assembled a list of 163 genes that demonstrated expression bias in favor of the *M. guttatus* allele as described above (**Tables S8** and **S9**). *M. guttatus*-bias could be the result of cis-regulatory allele-specific expression bias or imprinting (Stupar and Springer, 2006; Zhang and Borevitz, 2009); the former would be *M. guttatus*-biased regardless of the crossing direction. We validated 7 of these putatively *M. guttatus-*biased genes as above and all are expressed in endosperm. Four genes were imprinted, exhibiting paternally-biased expression in isolated endosperm regardless of the direction of the cross (**Table 2**, **Figure 6**). Intriguingly, only one of the validated genes exhibits temporally significant expression changes: Migut.E01117 is unexpressed until 6 DAP then declines at 8 DAP; the remaining were not identified as DEGs by our time series analysis. All four genes have imprinted homologues in other species (**Table 2**). The remaining three putative *M. guttatus*-biased genes exhibited allele-specific expression (**Table 2**).

**Table 2:** Genes exhibiting *Mimulus guttatus* allele specific bias selected for validation.

*M. guttatus* gene name	*A. thaliana* homologue	Gene name	Description	Imprinted	Imprinted homologues
Migut.E01117	AT5G52230	*MBD13*	Methyl-CpG binding transcription regulator	Yes	*Capsella rubella* (MEG and PEG), maize (PEG), castor (MEG)
Migut.H00744	AT5G09790	Trithorax-related 5 (*ATXR5*)	Histone-lysine N-methyltransferase: targets H3K27 for epigenetic silencing of repetitive and transposon elements	Yes	Maize (PEG), *Solanum chilense* (PEG), and *S. peruvianum* (PEG)
Migut.I00545	AT5G53150	*DnaJ*	DnaJ heat shock N-terminal domain chaperone protein	Yes	*Capsella grandiflora* (PEG)*, Arabidopsis thaliana* (PEG)*, S. peruvianum, S. chilense* (MEG) and *S. arcanum* (PEG), maize (MEG), sorghum (MEG), castor (PEG)
Migut.I00995	AT1G51730	RWD domain-containing protein	Ubiquitin-conjugating enzyme family protein	No	N.A.
Migut.M00083	AT5G08650	WD-repeat protein	RanBPM protein, function unknown	No	N.A.
Migut.N01317	AT2G16730	Beta-galactosidase 11-related (*BGAL11*)	Catalysis of the hydrolysis of terminal, non-reducing beta-D-galactose residues in beta-D-galactosides.	Yes	Rice (MEG), *S. chilense* (MEG and PEG)
Migut.L01896	AT3G05860	*AGL45*	MADS-box transcription factor	No	N.A.

Imprinted expression was confirmed by rt-PCR, enzymatic digestion, and Sanger sequencing. References: Capsella: (Hatorangan et al., 2016; Lafon-Placette et al., 2018); maize: (Waters et al., 2013); Arabidopsis thaliana: (Pignatta et al., 2014); Solanum: (Florez-Rueda et al., 2016; Roth et al., 2018); sorghum: (Zhang et al., 2016); rice (Luo et al., 2011; Chen et al., 2018); castor (Xu et al., 2014). ASE, allele specific expression. We have designated as N.A. genes that were not imprinted in Mimulus; we did not search for homologues of these genes in other taxa.

## Discussion

Seed development can be partitioned into stages: early embryo patterning and endosperm proliferation, embryo organ initiation and morphogenesis, and the onset of maturation, during which the endosperm accumulates seed storage proteins and the embryo enters dormancy (Agarwal et al., 2011). In *Mimulus*, as in *Arabidopsis*, the majority of genes expressed in ovules and seeds are expressed across all stages of ovule and seed development and comparatively few genes are *stage-specific* with an even smaller number *exclusive* to ovules or seed development from 2 to 8 DAP (**Table S7**), suggesting that the majority of genes fulfill multiple developmental and biological roles (Le et al., 2010; Belmonte et al., 2013; Chen et al., 2014).

A question of particular interest is “what is the set of genes that regulate the *ab initio* cellular type of endosperm development and furthermore, are the expression of these key regulators uniquely expressed in cellular endosperms”? We observe some overlap between the set of genes expressed in a *seed-exclusive* manner in *M. guttatus* and *seed-exclusive* genes detected in other species (i.e., *A. thaliana*, maize, and tomato). Additionally, there are 8 *seed-exclusive* tomato genes (associated with 25 *seed-exclusive Mimulus* homologues) that are not expressed in a seed exclusive fashion in maize or *Arabidopsis*. These genes might be good candidates for future functional analyses that aim to identify regulatory differences between *ab initio* cellular and syncytial endosperm developmental strategies. However, it remains possible that the difference between *ab initio* and syncytial endosperm development is determined by differences in structural aspects of encoded proteins or in other changes (e.g., post-transcriptional) that are not reflected in the transcriptional patterns that we have identified in this work. Only future functional analyses can address this question. We do note a lack of overlap between *A. thaliana seed-exclusive* and maize *seed-exclusive* genes enriched in endosperm but unexpressed in *M. guttatus* seeds. This suggests a significant degree of transcriptional divergence between these two taxa that both display syncytial development and may reflect the fact that these species are highly genetically and evolutionarily divergent and show marked differences in seed development overall (*A. thaliana* is a dicot and maize is a monocot).

Most expressed genes do not exhibit significant changes associated with fertilization and major developmental events within seeds. A substantial portion, however, are differentially expressed and segregate into clusters that are suggestive of their roles in development. Three co-expression gene clusters (clusters 2, 17, and 18) deserve further discussion here due to their associated functional GO-terms and the presence of stage-specific gene expression patterns.

### Cluster 17: Genes Enriched for Mitotic Cell Cycle and Chromatin Organization

Genes within cluster 17 are highly expressed in ovules, decline in expression and then increase in globular- and heart-stage seeds (**Figure 3**). Overrepresented GO-terms indicate functional roles in the regulation of chromatin organization, DNA replication and the cell cycle. A *Mimulus* homologue of *TOPOISOMERASE II* (*TOPII*), which functions to ensure proper chromosome movement during cell division in *A. thaliana* (Martinez-Garcia et al., 2018) is also contained within cluster 17. Other genes within this cluster have homologues associated with chromatin organization including At*CMT3* and At*MET1*, which act to maintain methylation of CHH and CG sites, respectively, during DNA replication (Lindroth et al., 2001; Kankel et al., 2003). In *A. thaliana*, seed development is accompanied by extensive gain of CHH methylation, especially within transposable elements, and *CMT3* and *MET1* transcription is largely confined to the embryo (Hsieh et al., 2009; Kawakatsu et al., 2017). By contrast, multiple homologues of *CMT3* are expressed in tomato endosperm at the globular-stage (Roth et al., 2019), raising the possibility that the function of *CMT3* in *Mimulus* is more similar to tomato than *A. thaliana*. Also within this cluster is a *Mimulus* homologue of At*HMGB3*, a family of proteins with an HMG-box DNA-binding domain that act as global modulators of chromatin structure through the assembly of nucleoprotein assemblies (Pedersen and Grasser, 2010). We also find a homologue of *MINICHROMOSOME MAINTENANCE 7* (*MCM7*), and the timing of its expression mirrors its expression pattern in *A. thaliana.* At*MCM7* is part of a complex which unwinds DNA prior to replication, and is maternally expressed in the egg and central cells of the ovule, then biparentally expressed the proliferating embryo and endosperm (Herridge et al., 2014).

### Cluster 2: Genes Associated With the Recruitment of a Secondary Cell Wall Biosynthesis Regulatory Module

Cluster 2 is comprised of genes that are minimally expressed from 0 to 4 DAP, peak dramatically in globular-stage seeds (6 DAP), and then again decline in heart-stage seeds (8 DAP) (**Figure 3**). These genes show a strong overrepresentation of GO-annotations associated with secondary cell wall biogenesis and cell wall remodeling activities (**Table S5**). Cluster 2 contains *Mimulus* homologues of the *Arabidopsis IRREGULAR XYLEM* (*IRX*) genes including *IRX1* (At4g18780), *IRX3* (At5g17420), and *IRX5* (At5g44030), all encoding cellulose synthase catalytic subunits required for secondary wall formation (Taylor et al., 1999; Taylor et al., 2000; Taylor et al., 2004), as well as *Mimulus* homologues of *IRX7* (At2g28110), *IRX9* (At2g37090), *IRX10* (At1g27440), *IRX12* (At2g38080), *IRX14* (At5g67230), and *IRX15-L* (At5g67210), which encode additional enzymes involved in secondary cell wall synthesis (Zhong and Ye, 2015). Cluster 2 also contains transcription factors that constitute a key regulatory network of secondary cell wall formation. These include *Mimulus* homologues of *SECONDARY WALL-ASSOCIATED NAC DOMAIN PROTEIN 2* (*SND2*) (At4g28500), *KNAT7* (At1g62990), and five members of a MYB family of transcription factors that regulate secondary cell wall formation (At4g22680, At3g08500, At4g33450, At1g73410, At1g66230). Thus, a large portion of the regulatory machinery underlying secondary cell wall synthesis in *Arabidopsis* appears to be conserved in *Mimulus* and is recruited to mediate some secondary cell wall events specific to this stage of development, which includes rapid proliferation of endosperm (Oneal et al., 2016). In addition to the large number of genes with known functional annotations in *Arabidopsis*, many other genes within the expression cluster would be expected to regulate and function in secondary cell wall biosynthesis in *Mimulus*. As such, our analysis identifies additional *Mimulus* candidates for future functional studies of secondary cell wall regulation.

### Cluster 18: Genes Involved in Auxin Synthesis and the Accumulation of Nutrient Reservoirs in Endosperm

The plant hormone auxin is associated with nearly every aspect of plant growth and development, including seed and fruit development (Zhao, 2010; Li et al., 2016; Figueiredo and Köhler, 2018). We detected the expression of several components of the auxin biosynthesis and signaling pathway in cluster 18 which peaks in 8 DAP seeds (**Figure 3**). Indeed, two such genes expressed in *exclusively* in heart-stage seeds are *M. guttatus* homologues of *TRYPTOPHAN AMINOTRANSFERASE RELATED2* (*TAR2;* Migut.D01989) and *YUCCA4 (YUC4;* Migut.A00287*)* which encode the TAR and YUCCA enzymes in the major auxin biosynthetic pathway (Brumos et al., 2014). Multiple *AUXIN RESPONSE FACTOR* (*ARF*) genes are also expressed, including three homologues of the transcription factor *ARF19* (Migut.L01587, Migut.J00834, Migut.L01914), which in tomato is highly expressed in seed coat (Pattison et al., 2015) and can play a major role in regulating seed size (Sun et al., 2017).

*Arabidopsis* homologues of *Mimulus* genes found in this cluster are associated with seed oil biosynthesis, and display increasing expression within maturing *A. thaliana* seeds (At5g52920, At3g02630, and At5g63380) (Hajduch et al., 2010). Thus, we suggest these *Mimulus* genes play a role in the metabolic processes which generate the energy stores required by the mature seed phenotype. One such group of major regulators of seed maturation includes the *LEAFY COTYLEDON* (*LEC*) group of genes. *FUS3* is a component of this group and acts as a transcriptional activator (Keith et al., 1994; Luerßen et al., 1998; Yamamoto et al., 2009; Wang and Perry, 2013); *fus3* mutants lose embryo identity and fail to germinate (Harada, 2001). *ABI3* is another transcriptional activator, and *abi3* mutants exhibit impaired embryo maturation and germination (Parcy et al., 1994). Both *FUS3* (Migut.F00019/At3g26790) and *ABI3* (Migut.D00511/At3g24650) segregate into cluster 18, and are differentially expressed over the course of seed development: Mg*FUS3* is expressed solely in heart-stage seeds, while Mg*ABI3* is expressed in globular- and heart-stage seeds. Similarly, the nearest homolog of *LEC1-like* (At*L1L*/At5g47670/Migut.M01289) is also contained with this cluster. These results suggest that Mg*ABI3* and Mg*FUS3* may regulate the expression of this cluster of genes in a manner similar to what is seen in *Arabidopsis,* maize, barley, and tomato seeds (McCarty et al., 1991; Bassel et al., 2006; Abraham et al., 2016).

### *Mimulus* MADS-Box Genes Expressed During Seed Development

MADS-box genes are transcription factors that play key roles in regulating developmental transitions involved in germination, root growth, flowering, and reproduction (Zhang and Forde, 1998; Saedler et al., 2001; Bemer et al., 2010). Surveys of type I MADS-box expression in *A. thaliana* have found that members of this gene family are expressed in multiple cell types within the female gametophyte and the developing seed (Bemer et al., 2010; Zhang et al., 2018). The origin of type II MADS-box genes can be attributed largely to whole genome duplication events (Shan et al., 2007), while type I MADS-box genes appear to have evolved *via* both whole genome duplication and smaller scale duplication events (Nam et al., 2004). Prior work has found widespread evidence of gene loss, subfunctionalization, and neofunctionalization of this important gene family (Vandenbussche et al., 2003; Kramer et al., 2004; de Martino et al., 2006; Kater et al., 2006; Airoldi and Davies, 2012).

It is intriguing that several temporally co-expressed type I MADS genes are each other’s nearest relatives (**Figure 5**). For example, two genes that peak in expression in ovules (Migut.B00480 and Migut.N01504) and two that peak in 2 DAP seeds (Migut.B00708 and Migut.D01476) are sister to each other in the NJ tree (**Figure 5**) and fall into the same expression clusters (clusters 5 and 1, respectively). Together with Migut.B00479, these genes are contained within a clade that includes the MADS genes At*AGL23* (At1g65360), At*AGL28* (At1g01530), and At*AGL62* (At5g60440). In *A. thaliana*, these genes function in regulatory networks in the female gametophyte and developing seed (Colombo et al., 2008; Kang et al., 2008; Steffen et al., 2008; Bemer et al., 2010; Figueiredo et al., 2015; Fiume et al., 2017; Zhang et al., 2018). Two MADS genes that are differentially expressed in 6 DAP seeds (Migut.B01089 and Migut.L01896) are contained within a clade that includes At*AGL96* and At*AGL48*, both expressed in the globular embryo (Bemer et al., 2010), as is the closely related tomato MADS-box gene Solyc12g016150 (Pattison et al., 2015). Migut.K01032 and Migut.H01211 are also paired and peak in 6 DAP seeds.

Several type I MADS-box genes exhibit temporal patterns of expression in *M. guttatus* that mirror their expression in *A. thaliana*. Four MADS-box genes are expressed in the female gametophyte but not the fertilized seed of *A. thaliana*, including At*AGL49* (At1g60040), At*AGL60* (At1g72350), At*AGL73* (At5g38620), and At*AGL83* (At5g49490) (Bemer et al., 2010). *M. guttatus* homologues of these genes (At*AGL49*: Migut.B01668; At*AGL60, AGL73*, and *AGL83*: Migut.N01504) are also expressed in *M. guttatus* ovules but not in seeds (**Table S7**). Similarly, At*AGL102* (At1g47760), At*AGL34* (At5g26580), and At*AGL90* (At5g27960) are expressed in *A. thaliana* seeds but not prior to fertilization (Bemer et al., 2010; Zhang et al., 2018); the *M. guttatus* homologues of these genes *(*At*AGL102*: Migut.K00930; At*AGL34* and At*AGL90*: Migut.B01455) are also unexpressed in ovules, peaking dramatically in four-cell embryo *M. guttatus* seeds (2 DAP) (**Table S7**). Moreover, six of the seed-specific genes we identified are type I MADS-box genes, including one that is also seed-specific in *A. thaliana* (At*AGL45*/At3g05860: Migut.B01089). Finally, At*AGL26* (At5g26880), At*AGL28* (At1g01530), At*AGL40* (At1g01530), and At*AGL62* (At4g36590) are expressed both in the *A. thaliana* female gametophyte and the seed, as are their *M. guttatus* homologues (At*AGL26:* Migut.N01481; At*AGL28*, At*AGL40,* and At*AGL62*: Migut.B00480). While the history of duplication, retention and loss of type I MADS-box genes in *Mimulus* remains uncertain, these findings are suggestive of shared roles for these important transcription factors in gametophytic and seed development in *A. thaliana* and *M. guttatus*.

### Paternally Imprinted Genes in *Mimulus* Endosperm

The prevailing hypothesis for the evolution of imprinted expression is the kinship theory, which posits that biases in the expression of maternally or paternally derived genes have evolved to maximize fitness of mothers *vs*. offspring with respect to offspring provisioning *via* endosperm (Haig and Westoby, 1989; Haig, 2004). Under this scenario, genomic conflict over maternal resources could lead to rapid evolution of imprinted loci, resulting in rapid sequence divergence and/or turnover in the imprinting status of genes among even closely related species (Köhler et al., 2010; Köhler et al., 2012). In general, surveys of imprinted genes have found little overlap in imprinting status of loci between closely related (Wolff et al., 2011) or distantly related taxa (Luo et al., 2011; Waters et al., 2013; Yoshida et al., 2018), however, some loci are imprinted in several different species. Here we report the first validated imprinted genes in *M. guttatus* [(but see (Kinser et al., 2018)]. We found that all four of our validated paternally imprinted genes (PEGs) have homologues that are imprinted in other species, including maize, sorghum, and rice (**Table 2**). Given our *ad hoc* method for validating candidate PEGs, which included as a criterion the potential for imprinting in other taxa, this is not surprising. Nevertheless, the fact that these genes are imprinted in multiple species, including one with imprinted homologues in six other species (Migut.I00545: *DnaJ* heat shock chaperone protein), suggests that proteins encoded by these genes perform similar functions in endosperm across plant taxa.

Previous work indicates that many PEGs are involved in epigenetic regulation (Waters et al., 2013; Pignatta et al., 2014) *via* DNA methylation or repressive histone modifications. Two *M. guttatus* genes we identified as exhibiting paternally-biased expression belong to these functional categories: a methyl-CpG binding transcription factor Migut.E01117 (At*MBD13*/At5g52230) and a chromatin remodeling protein, Migut.H00744 (At*ATXR5/*At5g09790). Methyl-CpG binding transcription factors mediate CpG methylation by coordinating the activities of histone deacetylases and histone methyltransferases (Zemach and Grafi, 2007). Likewise, At*ATXR5* promotes gene silencing at constitutive heterochromatin and repression of TEs by orchestrating mono-methylation at H3K27 (Jacob et al., 2009). Homologues of both genes are imprinted in *Capsella*, maize, and castor (*MBD13*) and in maize and two species of *Solanum (* Waters et al., 2013; Xu et al., 2014; Hatorangan et al., 2016; Roth et al., 2018 *).* Homologues of *DnaJ* heat shock protein (Migut.I00545) are imprinted in six other genera, including *Solanum* (Waters et al., 2013; Xu et al., 2014; Hatorangan et al., 2016; Roth et al., 2018), while homologues of *BGAL11* (Migut.N01317) are imprinted in rice and *Solanum* (Luo et al., 2011; Chen et al., 2018; Roth et al., 2018).

Disruption in the allelic dosage of paternally and maternally imprinted genes in endosperm can lead to dysfunctional endosperm development and ultimately, seed abortion, and is associated with hybrid seed failure (Haig and Westoby, 1989; Haig and Westoby, 1991; Scott et al., 1998; Kinser et al., 2018). Postzygotic isolation *via* such hybrid seed inviability is widespread in *Solanum* (Baek et al., 2016), and imprinted loci have been implicated in this process (Florez-Rueda et al., 2016; Roth et al., 2018; Roth et al., 2019). It is striking that three of our validated PEGs have homologues that are imprinted in *Solanum* (**Table 2**), and is suggestive of shared patterns of imprinting in these taxa, which both exhibit a cellular endosperm development (Arekal, 1965; Oneal et al., 2016). Hybrid seed inviability is also common in the *M. guttatus* complex (Vickery, 1966; Vickery, 1978) and is known in some cases to be caused by a failure of endosperm development (Oneal et al., 2016; Coughlan et al., 2018). Moreover, the effects of some genomic loci contributing to hybrid seed inviability is dependent upon whether they were contributed by the maternal or paternal genome (Garner et al., 2016). Future, systematic surveys of gene expression in isolated endosperm of reciprocally-crossed endosperms in *M. guttatus* and in closely related, diploid sister species (e.g., *Mimulus nudatus, Mimulus tilingii,* and *Mimulus decorus*), coupled with characterizations of their expression in incompatible hybrids could demonstrate whether genomic imprinting, which is thought to evolve *via* parental conflict over maternal resources, is a major factor driving hybrid incompatibility and speciation in this rapidly evolving group.

In conclusion, we used RNA sequencing to profile dynamic gene expression events in seeds across an 8-day time course and used gene ontology enrichment analyses to reveal statistically overrepresented biological functional categories of co-expressed clusters of genes. With respect to the regulation of a number of important biological events during seed development (e.g., secondary cell wall biosynthesis, seed maturation, auxin synthesis) the coexpressed gene clusters reported here provide valuable entry points for the investigation of novel molecular regulatory mechanisms during *Mimulus* seed development. Similarly, our analysis of the *Mimulus* MADS type I transcriptional regulators highlights the subset of the family expected to function during seed development and furthers our understanding of the evolution of this gene family. Furthermore, we detected and validated genes exhibiting paternally imprinted expression. The conservation of the imprinted status of homologous genes within other studied angiosperms strongly indicates they play a functional role during endosperm development in *Mimulus*, an area for future study. Our data broadens the taxonomic framework underlying studies of seed development generally and constitutes a valuable resource to other researchers exploring seed development and hybrid inviability in *Mimulus*, as well as future studies of comparative gene expression.

## Data Availability Statement

DNA and RNA sequence data have been deposited in the SRA and GEO database (accessions SRX6435295, SRX6435296 (DNA) and GSE123424 (RNA)). Supporting data is provided in the supplementary tables with this submission and includes all primers used in this study, alignment statistics for RNA libraries, FPKM values for all genes, the results of edgeR, Next maSigPro, clustering, GO-term enrichment analyses, and raw SNP count data for genes exhibiting M. guttatus-biased expression from 2-8 DAP.

## Author Contributions

MF-V, EO and RF collaboratively designed the study. MF-V managed overall experimental approaches and generated RNA samples and libraries and validated imprinted genes. EO, MF-V, GV and ML analyzed the data. EO managed analysis approaches and assembled list of genes with *M. guttatus-*biased expression. MC performed the histological sectioning and analysis. CR generated seed morphology data. MF-V, EO and RF managed data interpretation and wrote the manuscript. SC and JW revised and edited the manuscript.

## Funding

This research was funded by NSF grants IOS-1557692 (RF) and IOS-1558113 (JW), as well as a grant from the European Union’s Horizon2020 research and innovation programme under the Marie Skłodowska-Curie grant agreement (No 690946) to SC and RF. CR’s participation in this project was funded by the Integrated Molecular Plant Systems NSF-REU (Award DBI-1558579).

## Conflict of Interest

The authors declare that the research was conducted in the absence of any commercial or financial relationships that could be construed as a potential conflict of interest.
