# Supplementary material for: Developmental Analysis of Mimulus Seed Transcriptomes Reveals Functional Gene Expression Clusters and Four Imprinted, Endosperm-Expressed Genes
Source: Front Plant Sci. 2020 Feb 25;11:132. doi: 10.3389/fpls.2020.00132 (PMC7052496; doi:10.3389/fpls.2020.00132)
Supplement: Supplementary file 1 [file DataSheet_1.pdf]

Developmental analysis of *Mimulus* seed transcriptomes reveals functional gene expression clusters and four imprinted, endosperm-expressed genes

Supplementary Material

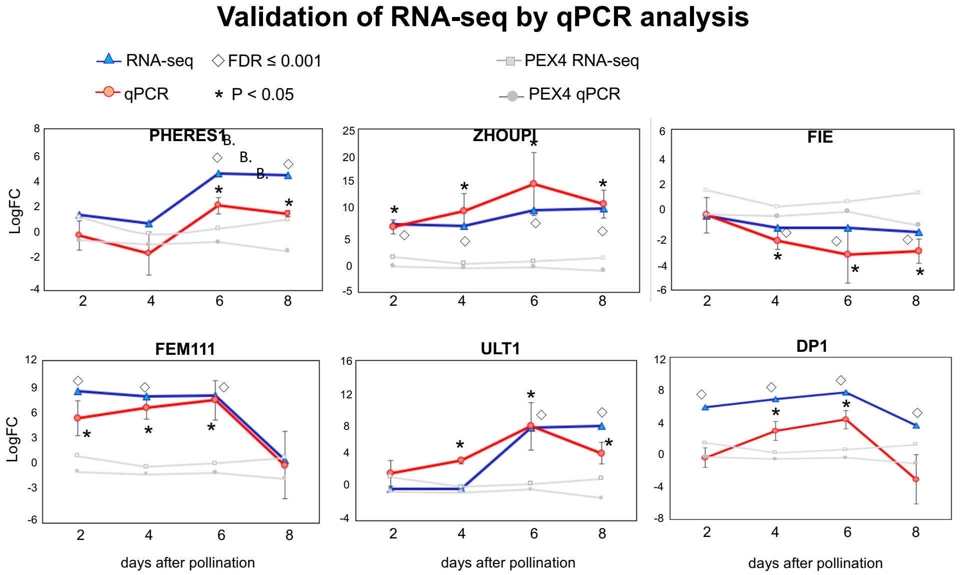

Supplementary Figure 1. Validation of RNA isolation, mapping and normalization of gene expression with quantitative real-time PCR (qRT-PCR) of independently-collected, triplicate RNA samples from unfertilized ovules and whole hybrid seeds (see Methods). The logFC was calculated relative to the level of expression observed in unfertilized ovules on a subset of genes expressed in 2-8 DAP samples including Migut.C01325 (At*PHERES1*/At1g65330), Migut.N01183 (At*ZHOUPI*/At1g49770), Migut.M01812 (At*FIE*/At3g20740), Migut.B01455 (At*FEM111*/At5g48670), Migut.E00987 (At*ULT2*/At2g20825), and Migut.A00696 (At*DP1*/At4g23690); the Migut.D02188 (AtEF1A/At1g07930) gene was used as a reference gene to normalize target gene transcript level amongst all samples. Transcription levels that exhibit a LogFC ≥ 2 or ≤ -2 with a FDR ≤ 0.01 (RNA-seq) or P < 0.05 (qRT-PCR) are indicated as a white diamond and an asterisk, respectively. Gray line with square or circle depict the low expression variance of PEX4 gene across all time points from RNA-seq and qRT-PCR data, respectively.

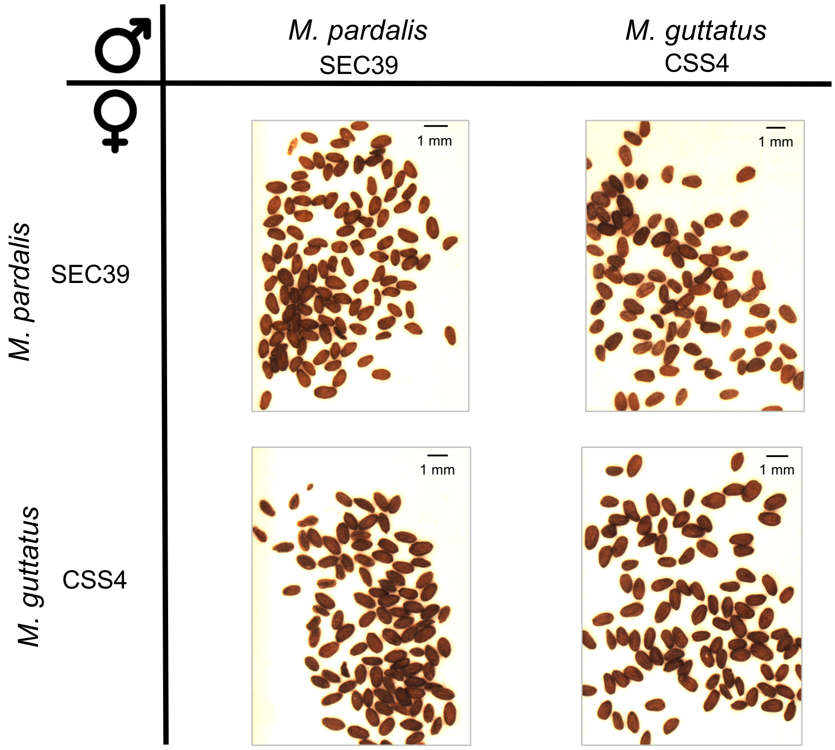

Supplementary Figure 2. Outer morphology of seeds from reciprocal *M. pardalis* x *M. guttatus* crosses.

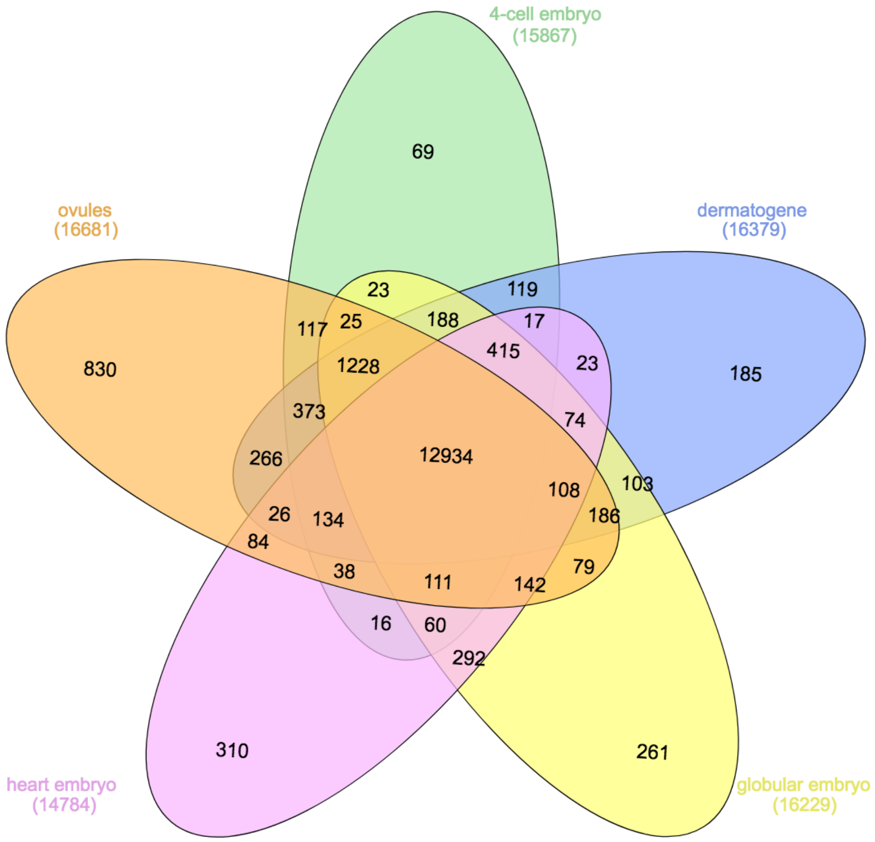

Supplementary Figure 3. Overlap of genes expressed in unfertilized ovules and developing seeds.

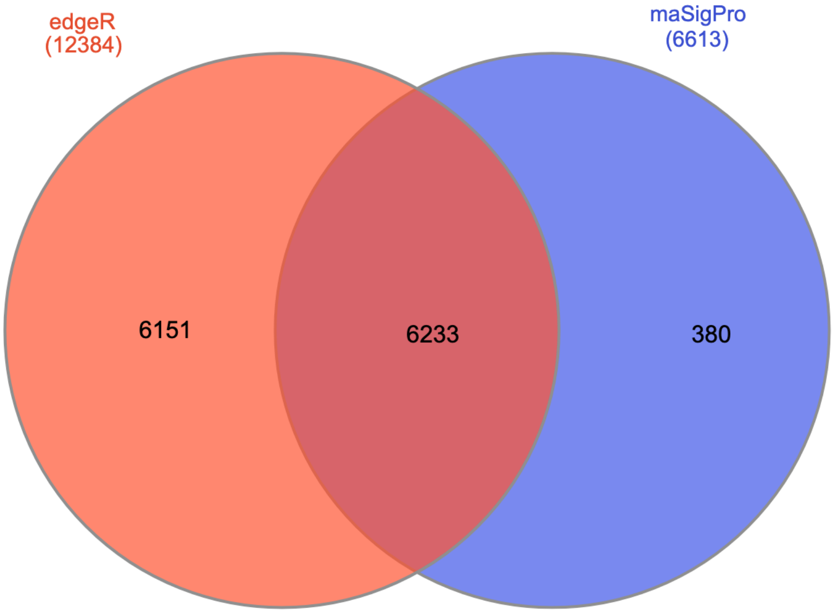

Supplementary Figure 4. Overlap of differentially expressed genes (DEGs) detected by edgeR and Next maSigPro.

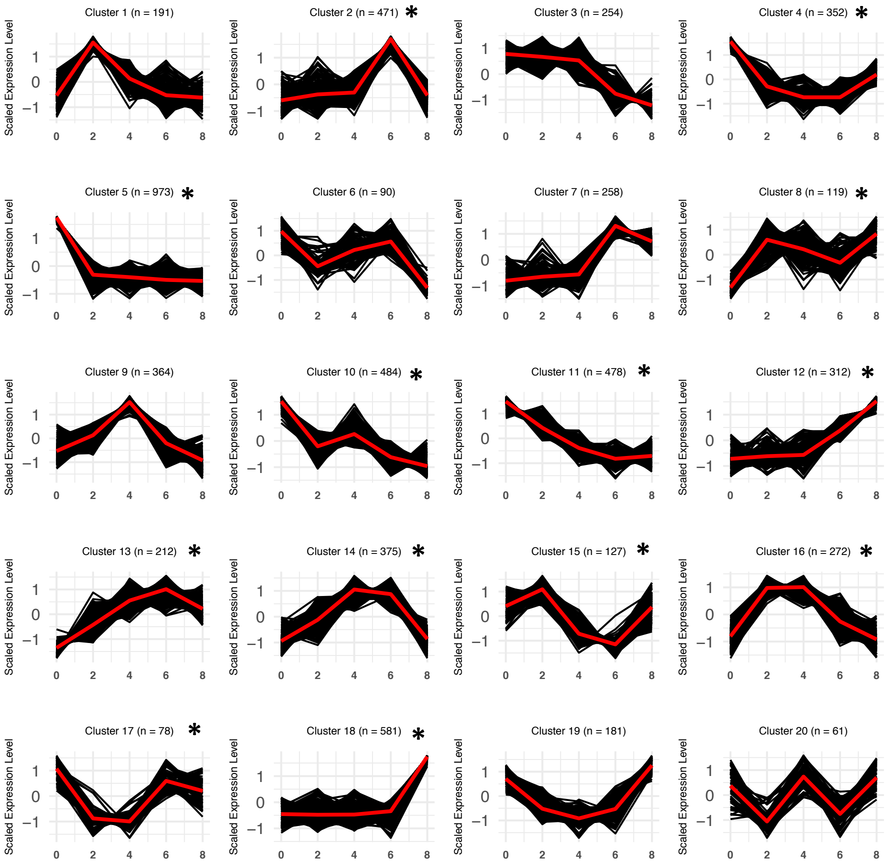

Supplementary Figure 5. K-means clusters of co-expressed genes and the number of genes assigned to each cluster. Clusters with significant GO term enrichment are indicated by an *.

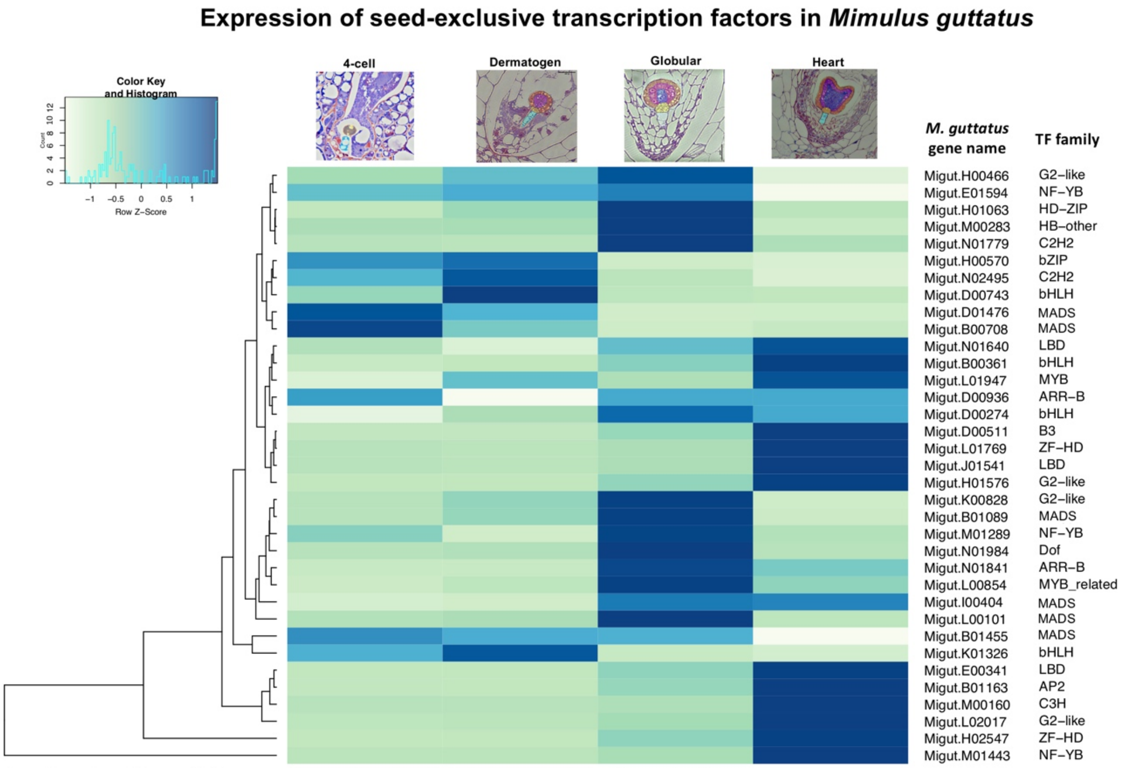

Supplementary Figure 6. Heatmap of expression of seed-exclusive transcription factors in *M. guttatus*

Supplementary Tables

Supporting Table 1. All primers used in this study.

Supporting Table 2. Alignment statistics for RNA libraries. Includes for each sample: raw RNA reads, reads remaining after quality trimming, number and percentage of uniquely mapped reads to each parental pseudo-reference, number and percentage of properly paired primary mapped reads in final RNA alignments, percentage reads mapping to IM62 reference genome, and the number of reads unambiguously assigned to annotated genes.

Supporting Table 3. Results of edgeR timecourse analysis, where multiple pairwise comparisons were made: 0 vs 2 DAP, 2 vs 4 DAP, 4 vs 6 DAP, and 6 vs 8 DAP. Differentially expressed genes are those with FDR ≤ 0.01.

Supporting Table 4. Results of Next maSigPro analysis of differentially expressed genes. Includes Benjamini-Hochberg adjusted p-values, R^2 of temporal correlation of gene expression, most similar *A. thaliana homologue*s, gene categories.

Supporting Table 5. Hartigan-Wong K-means clustering on the set of overlapping DEGs identified by edgeR and Next maSigPro. A. thaliana orthologs were determined as described in the Methods.

Supporting Table 6. Results of GO-term enrichment for 20 k-means clusters, filtered by REVIGO to remove redundant terms. P-values are Benjamini-Hochberg adjusted.

Supporting Table 7. *M. guttatus* genes whose expression is *specific* to different stages of development included in our study (i.e., unfertilized ovules and seeds collected from 2-8 DAP) and/or *M. guttatus* genes *exclusive* to ovules and seeds (see Methods), together with their nearest *A. thaliana* homologues. Genes with nearest homologs that are seed exclusive *A. thaliana*, maize, or domesticated tomato are noted.

Supporting Table 8. SNP counts at genes exhibiting *M. guttatus*-biased expression in at least one timepoint from 2-8 DAP.

Supporting Table 9. List of *Mimulus* genes exhibiting *M. guttatus*-biased expression in at least one timepoint from 2-8 DAP with their nearest *A. thaliana* homologues as determined by BLASTx.

Supporting Table 10. FPKM values of all genes with their most similar *A. thaliana* homologues and gene categories
